# Anti-dsDNA-NcX ELISA: dsDNA-loaded nucleosomes improve diagnosis and monitoring of disease activity in systemic lupus erythematosus

**DOI:** 10.1186/ar3250

**Published:** 2011-02-10

**Authors:** Robert Biesen, Cornelia Dähnrich, Anke Rosemann, Fidan Barkhudarova, Thomas Rose, Olga Jakob, Anne Bruns, Marina Backhaus, Winfried Stöcker, Gerd-Rüdiger Burmester, Wolfgang Schlumberger, Karl Egerer, Falk Hiepe

**Affiliations:** 1 Department of Rheumatology and Clinical Immunology, Charité Universitätsmedizin Berlin, Chariteplatz 1, Berlin D-10117, Germany; 2 EUROIMMUN Medizinische Labordiagnostika AG, Seekamp 31, Lübeck D-23560, Germany

## Abstract

**Introduction:**

The objective of this study was to compare the clinical usefulness of the new anti-double-stranded DNA nucleosome-complexed enzyme-linked immunosorbent assay (Anti-dsDNA-NcX ELISA), which is based on dsDNA-loaded nucleosomes as antigens, with established test systems based on dsDNA or nucleosomes alone for systemic lupus erythematosus (SLE) diagnostics and determination of disease activity.

**Methods:**

Sera from a cohort of 964 individuals comprising 207 SLE patients, 357 disease controls and 400 healthy donors were investigated using the Anti-dsDNA-NcX ELISA, Farr assay, Anti-dsDNA ELISA, Anti-nucleosome ELISA and *Crithidia luciliae *immunofluorescence (CLIF) assay, all of which are tests available from EUROIMMUN Medizinische Labordiagnostika AG (Lübeck, Germany). Receiver operating characteristic curve analyses were performed to compare the sensitivity and specificity of each assay. The test results yielded by these assays in a group of 165 fully characterized SLE patients were compared with the corresponding medical records.

**Results:**

The Anti-dsDNA-NcX ELISA was found to have a sensitivity of 60.9% and a specificity of 98.9% in all 964 individuals at the manufacturer's cutoff of 100 U/ml. At a comparable specificity of 99%, the sensitivity amounted to 59.9% for the Anti-dsDNA-NcX ELISA, 54.1% for the Farr assay, 53.6% for the antinucleosome ELISA and 35.8% for the anti-dsDNA ELISA. The CLIF assay had a sensitivity of 28.0% and a specificity of 98.2%. The Anti-dsDNA-NcX ELISA correlated mostly with global disease activity in a cross-sectional analysis. In a longitudinal analysis of 20 patients with 69 patient visits, changes in Anti-dsDNA-NcX ELISA and antinucleosome ELISA results correlated highly with changes in disease activity over time.

**Conclusions:**

The use of dsDNA-complexed nucleosomes as antigens in ELISA leads to optimized determination of diagnosis and disease activity in SLE patients and is available for clinical practice.

## Introduction

Systemic lupus erythematosus (SLE) is a chronic, relapsing, inflammatory autoimmune disease that mostly affects women of childbearing age. The disease is characterized by a diverse array of clinical findings and the overriding importance of autoantibodies against a wide range of self-antigens [1,2]. The hallmark of SLE, antibodies against double-stranded DNA (dsDNA), was described over 50 years ago and is usually regarded as an important serologic marker in the diagnosis and determination of disease activity [3,4]. These antibodies are commonly detected by using one of three different test systems: enzyme-linked immunosorbent assays (ELISA), radioimmunoassay (RIA; also known as a Farr assay) and the *Crithidia luciliae *immunofluorescence (CLIF) assay [4]. There are large differences in terms of the sensitivity and specificity of these tests, most notably among the commercial variants of anti-dsDNA ELISA.

In cases of elevated anti-dsDNA titers, it is clinically relevant to exclude other causes, such as infection with Epstein-Barr virus or hepatitis B virus as well as the use of drugs such as hydralazine, tumor necrosis factor (TNF) inhibitors, interferons, sulfasalazine and many more to ensure the accurate diagnosis of SLE [5,6]. Once the diagnosis of SLE is made, periodic measurements are considered essential to assess disease activity because an increase or even a decrease in anti-dsDNA antibody titers can predict a flare [7,8]. Adding to the uncertainty of determining disease activity, a recent study comprising a large number of patient visits reported no correlation with disease activity [9].

However, using pure dsDNA as a binding substrate in an anti-dsDNA ELISA remains a laboratory artefact. *In vivo *dsDNA bound to nucleosomes appears on blebs of apoptotic cells that are not immediately removed and is consequently presented to the immune system [10,11]. In recent years, it has become evident that nucleosomes containing dsDNA are the major T- and B-cell immunogens in patients with SLE [12,13]. Chabre *et al*. [14] and Amoura *et al*. [15] demonstrated that anti-dsDNA antibodies are always associated with antinucleosome antibodies (ANuA), but not vice versa, and that ANuA are exhibited prior to anti-dsDNA antibodies. So, it also became clear that the mass of anti-dsDNA antibodies and antihistone antibodies do not have distinct antibody specificity, but are subtypes of a whole family: ANuA [14,16,17].

In our initial study [12], ANuA were not present exclusively in SLE as they were also found in systemic sclerosis (SSc). Later we discovered that the antigen Scl-70 (topoisomerase I) is responsible for antinucleosomal antibody positivity in SSc and could further prove the negativity of a new, second-generation antinucleosome ELISA using purified nucleosomes free of Scl-70 in 119 sera of patients with SSc [18].

Up to now, nearly all commercially available anti-dsDNA ELISA kits have used protamine sulfate or poly-L-lysine as linkers to attach dsDNA to the plates. To minimize nonspecific reactions and to potentially mimic the type of dsDNA presentation *in vivo*, we used the strong adhesivity of nucleosomes to attach dsDNA to the solid phase for the first time. We thereby created a new ELISA, which we called Anti-dsDNA-NcX ELISA (an abbreviated name for anti-double-stranded DNA nucleosome-complexed ELISA).

Herein we compare the clinical significance of this novel Anti-dsDNA-NcX ELISA with previously established systems such as the Anti-dsDNA ELISA, Anti-nucleosome ELISA (anti-Nuc ELISA), CLIF and the gold standard for confirmation of SLE diagnosis, the Farr assay. We demonstrate that the Anti-dsDNA-NcX ELISA is an excellent nonradioactive test system to determine the diagnosis and disease activity of patients with SLE.

## Materials and methods

### Study participants

A total of 964 participants consisting of 564 patients and 400 healthy donors were studied from January 2004 to June 2007. Of this total, 207 patients had SLE according to the updated and revised classification criteria of the American College of Rheumatology (ACR) [19,20]. Demographic data and a detailed characterization of the SLE patients are shown in Table S1 in Additional file 1.

The non-SLE cohort included 162 individuals with rheumatoid arthritis (RA) [21], 88 patients with Sjögren's syndrome (SS) who fulfilled the revised European classification criteria [22], 81 patients with SSc according to the ACR criteria of 1980 [23] and 26 patients with myositis [24].

All patients were recruited from the Department of Rheumatology and Clinical Immunology, Charité University Hospital, Berlin, Germany. The Ethics Committee of the Medical Faculty of the Charité University Hospital approved the study, and written informed consent was obtained from all participants.

Sera from healthy donors were recruited in cooperation with the University of Lübeck and were investigated using the anti-dsDNA ELISA, antinucleosome ELISA and the Anti-dsDNA-NcX ELISA. The female:male ratio of healthy donors was 13:87, and their mean age was 35.13 years (age range, 18 to 65 years). Written informed consent was obtained from all healthy participants.

### Anti-dsDNA-NcX ELISA

The Anti-dsDNA-NcX ELISA microtiter plates (Nunc, Roskilde, Denmark) were coated at 4°C first with a 0.1 μg/ml concentration of an ultrapure nucleosome preparation from calf thymus (free of Scl-70, histone H1 and other nonhistone components) [18] in sodium carbonate buffer for 3 hours, followed by a 1.5 μg/ml concentration of highly purified, native dsDNA isolated from calf thymus in sodium carbonate buffer overnight. After being washed with 0.05% phosphate-buffered saline (PBS)-Tween 20 (vol/vol) and blocked for 2 hours with 0.1% PBS (wt/vol) casein, sera diluted 1:200 in 0.1% PBS (wt/vol) casein were added and allowed to react for 30 minutes. Bound antibodies were detected by use of antihuman immunoglobulin G peroxidase conjugate (EUROIMMUN Medizinische Labordiagnostika AG) and stained with tetramethylbenzidine (EUROIMMUN Medizinische Labordiagnostika AG) for 15 minutes. All steps were performed at room temperature. The optical density was read at 450 nm using an automated spectrophotometer (Spectra Mini; Tecan, Crailsheim, Germany). A highly positive index patient serum was used to generate a standard curve consisting of three calibrators (10, 100 and 800 international units (IU)/ml). IU were calculated for all samples using this three-point standard curve. The cutoff was optimized either by receiver operating characteristic (ROC) curve analysis (maximal sum of sensitivity plus specificity) or by predefined specificities of 98% and 99%. Commercially available anti-dsDNA ELISA, antinucleosome ELISA, CLIF and Farr assays (all from EUROIMMUN Medizinische Labordiagnostika AG) were used as reference assays and were performed according to the manufacturer's instructions.

### Statistics

The global reactivity of Anti-dsDNA-NcX ELISA and the diagnostic significance of the tests were assessed by ROC curve analysis, and the areas under the curve (AUC) were calculated using GraphPad Prism 5 software (GraphPad, La Jolla, CA, USA). Statistical analysis regarding autoantibody test results and disease variables obtained from medical records were calculated using the Mann-Whitney *U *test in SPSS version 16 software (SPSS, Inc., Chicago, IL, USA). Correlation of global disease activity according to the modified Systemic Lupus Erythematosus Disease Activity Index (mSLEDAI 2000) [25] with antibody assay titers was calculated using Spearman's rank order correlation (*r*_*s*_) test in GraphPad Prism 5 software. Linear regression analysis was used to assess the significance of correlations for changes in disease activity and biomarkers over time. *P *< 0.05 was considered statistically significant.

## Results

### Reactivity of Anti-dsDNA-NcX ELISA

**Figure 1:**
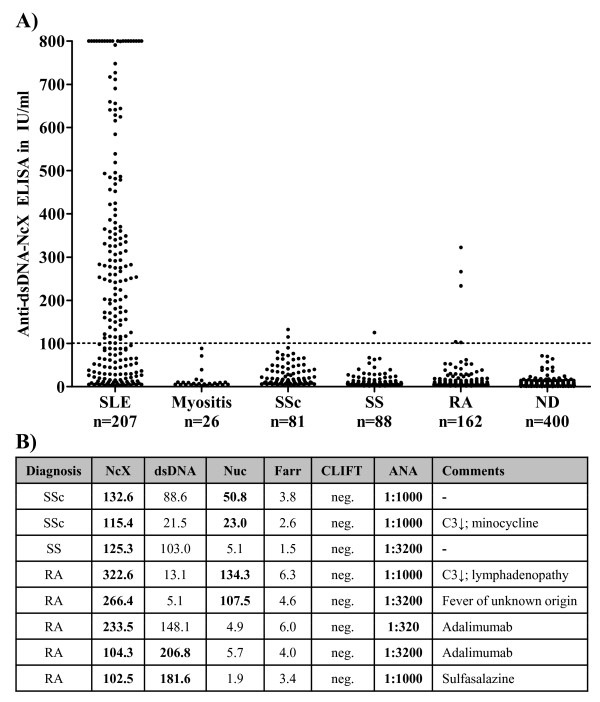
**Right-positive and false-positive test results of anti-double-stranded DNA nucleosome-complexed enzyme-linked immunosorbent assay (Anti-dsDNA-NcX ELISA)**. **(A) **Scatterplot showing Anti-dsDNA-NcX ELISA immunoglobulin G results in 964 different sera. Dotted line represents the manufacturer's threshold (100 IU/ml). Values >800 IU/ml were set to 800 IU/ml for a clearer arrangement of the figure. SLE, systemic lupus erythematosus; SSc, systemic sclerosis; SS, Sjögren's syndrome; RA, rheumatoid arthritis; ND, normal donors; **(B) **Table showing all non-SLE patients who tested positive in the Anti-dsDNA-NcX ELISA listed with the test results of all measured assays and clinically relevant findings. Positive test results according to a comparable specificity of 98.9% are marked in bold. Nuc, anti-dsDNA-nucleosome ELISA; Farr, radioimmunoassay; CLIF, *Crithidia luciliae *immunofluorescence assay; ANA, antinuclear antibodies; C3, complement component 3.

To assess the reactivity of the novel Anti-dsDNA-NcX ELISA, sera of 207 SLE patients, 400 healthy donors and 357 patients with different rheumatic diseases relevant in the differential diagnosis of SLE were measured (Figure 1A). At the manufacturer's threshold of 100 U/ml, a sensitivity of 60.4% and a specificity of 98.9% were calculated for the diagnosis of SLE. The results for eight disease controls were false-positives. Six of these eight non-SLE patients being positive in Anti-dsDNA-NcX had either positive anti-dsDNA or antinucleosome ELISA results at a specificity of 98.9% (Figure 1B). Notably, none of these eight SLE patients tested positive in the CLIF or Farr assay.

Out of interest, the medical records of all non-SLE patients who tested positive in the Anti-dsDNA-NcX ELISA were checked (Figure 1B). In five patients, causes other than SLE with the potential to induce anti-dsDNA antibodies were found, namely, drugs and fever indicating an unknown infection. Combining Anti-dsDNA-NcX test results and medical history with consequent exclusion of these five patients would lead to an increased specificity of 99.5% at the manufacturer's threshold.

### Analysis of test criteria

To further assess the performance criteria of the Anti-dsDNA-NcX ELISA with those of other assays for measuring antibodies against dsDNA and/or nucleosomes, the new ELISA was compared with the anti-dsDNA ELISA, the antinucleosome ELISA (free of Scl-70 and histone H1) and the Farr assay in sera from 964 individuals by using ROC curve analysis (Table 1).

**Table 1:** Performance criteria in ROC analysis^a^

Criteria	Anti-dsDNA-NcX	Anti-dsDNA	Antinucleosome	Farr
Area under the curve	0.9133^b^	0.8455	0.9118	0.8823
95% confidence interval	0.89 to 0.94	0.81 to 0.88	0.89 to 0.94	0.85 to 0.91
Sensitivity at specificity of 95%	72.5%^b^	55.6%	67.2%	65.7%
(cutoff)	(>49.7)	(>58.8)	(>6.8)	(>5.1)
Sensitivity at specificity of 98%	66.7%^b^	43.0%	58.0%	55.6%
(cutoff)	(>71.5)	(>99.7)	(>11.1)	(>6.0)
Sensitivity at specificity of 98.15%^c^	66.7%^b^	41.5%	55.6%	55.6
(cutoff)	(>72.03)	(>105.8)	(>12.1)	(>6.0)
Sensitivity at specificity of 99%	59.9%^b^	35.8%	53.6%	54.1%
(cutoff)	(>103.4)	(>151.6)	(>14.8)	(>6.5)
Maximum sum of sensitivity + specificity	170.9^b^	159.1	168.7	162.1

^a^dsDNA, double-stranded DNA. Test criteria for the Anti-dsDNA-NcX, anti-dsDNA and antinucleosome enzyme-linked immunosorbent assays (ELISAs), as well as for the anti-dsDNA radioimmunoassay (also known as a Farr assay), were calculated using receiver operating characteristic (ROC) curve analysis based on test readings of 964 samples from 207 systemic lupus erythematosus (SLE) patients, 357 disease controls and 400 healthy donors. Outcome parameters of ROC curve analysis were diagnosis versus no diagnosis of SLE. ^b^Highest value over all four assays. ^c^This specificity was calculated for the *Crithidia luciliae *immunofluorescence (CLIF) assay.

To check whether the performance of a single test system was significantly better than another one, we additionally tested the reported AUC values for significant differences. This formal statistical comparison revealed that the Anti-dsDNA-NcX ELISA and the antinucleosome ELISA were significantly better than the anti-dsDNA ELISA (*p *= 0.0024 and *p *= 0.0029, respectively). The Farr assay was not significantly better than any other ELISA, nor was the opposite the case.

The Anti-dsDNA-NcX ELISA revealed superior results in all performance criteria. The CLIF assay was not integrated into ROC curve analyses because it is a semiquantitative test. However, a sensitivity of 28.02% at a specificity of 98.15% was separately calculated for the CLIF assay. To allow direct comparison, the sensitivities of all other test systems are also shown at a specificity of 98.15% in Table 1.

### Comparison of test systems in terms of reactivity and diagnosis determination

As considerable differences were obtained in ROC curve analysis, two clinically relevant questions were addressed to further elucidate the similarities (question 1) and differences (question 2) of the test systems used. First, how often are sera positive in one test and positive in another test system? Second, how often are sera negative in one test but positive in another test system? Answers to the second question would give physicians clues to how often they might miss the correct diagnosis by determination of disease-specific autoantibodies using only one test system.

**Figure 2:**
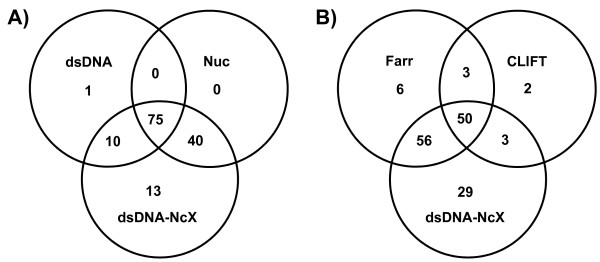
**Comparison of patient sera that tested positive in the anti-double-stranded DNA nucleosome-complexed enzyme-linked immunosorbent assay (Anti-dsDNA-NcX ELISA) and other investigated test systems shown in Venn diagrams**. **(A) **Absolute numbers of patient sera that tested positive in the Anti-dsDNA-NcX ELISA (dsDNA-NcX), the anti-dsDNA ELISA (dsDNA) and the anti-dsDNA nucleosome ELISA (Nuc) and their combined intersections are shown. **(B) **The same analysis as in Figure 2A is shown, including different detection techniques using Anti-dsDNA-NcX ELISA, Farr assay (radioimmunoassay) and *Crithidia luciliae *immunofluorescence (CLIF) assay is presented. Cutoffs for all positive test results were read out of receiver operating characteristic curve analysis at a specificity of 98.15% because this specificity was calculated for the CLIF assay.

To integrate the CLIF assay with its specificity of 98.15%, individual cutoffs of the other test systems at the same specificity (see also Table 1) were used. Intersections of the three ELISAs (Anti-dsDNA-NcX, antinucleosome and anti-dsDNA) and separately of dsDNA-NcX with the Farr and CLIF assays are illustrated in a Venn diagram shown in Figure 2. Of 207 sera, 139 were positive in all of the three ELISAs, which were compared at cutoffs read out at a specificity of 98.15%. Of these 139 positive sera, 99.28% were positive with the Anti-dsDNA-NcX ELISA, while 9.28% were exclusively positive with the Anti-dsDNA-NcX ELISA. In a comparison of the different detection techniques (Figure 2B), 149 sera were positive in the Farr assay, Anti-dsDNA-NcX ELISA or the CLIF assay. Exclusively positive sera were found as follows: two (1.3%) in the CLIF assay, six (4.0%) in the Farr assay and 29 (19.5%) in the Anti-dsDNA-NcX ELISA. Among these three tests, 138 (92.6%) of all 149 positive sera were positive in the Anti-dsDNA-NcX ELISA.

To answer the second question and to reveal differences between the assays, we further determined how often serum is negative in one test but positive in another test (Table 2). Clinically relevant for the verification of diagnosis, these frequencies indicate how often a diagnosis of SLE may be missed by using only one test.

**Table 2:** Frequency of sera being positive in one test system and negative in another test system^a^

SLE patients (*N *= 207)	NcX^+^ (*n *= 138)	dsDNA^+^ (*n *= 86)	Nuc^+^ (*n *= 115)	Farr^+^ (*n *= 115)	CLIF^+^ (*n *= 58)
NcX^- ^(*n *= 69)	-	1%	0%	13%	7%
dsDNA^- ^(*n *= 121)	44%	-	33%	35%	14%
Nuc^- ^(*n *= 92)	25%	12%	-	21%	8%
Farr^- ^(*n *= 92)	35%	14%	21%	-	5%
CLIF^- ^(*n *= 149)	57%	30%	43%	57%	-

^a^SLE, systemic lupus erythematosus; NcX, anti-dsDNA-nucleosome-complexed enzyme-linked immunosorbent assay (ELISA); dsDNA, double-stranded DNA; Nuc, anti-dsDNA-nucleosome ELISA; Farr, radioimmunoassay; CLIF, *Crithidia luciliae *immunofluorescence assay. Cutoffs for all test systems were read out of receiver operating characteristic curve analysis at a specificity of 98.15% to include the CLIF assay in that analysis. Frequencies of sera being positive in one selected test (column headings) and negative in another test system (left column stub headings) were filled to demonstrate the extent of cross-reactivity between tests. The positive sera were compared with the negative sera to calculate percentages. For example, among all anti-dsDNA-NcX ELISA-negative sera (*n *= 69), 1% were positive on the basis of the anti-dsDNA ELISA.

### Clinical associations of assays

To reveal clinical associations of investigated test systems, assay titers of patients with a distinct present clinical finding were compared with those of patients without that finding using the Mann-Whitney *U *test (Table 3). Clinical findings were read out of medical records consisting of ACR criteria, mSLEDAI 2000 score, laboratory parameters and immunosuppressants and/or antimalarials.

**Table 3:** Comparison of test assay titers in patients with versus those without a distinct present clinical finding^a^

		*P *value				
Disease feature	Number of patients	NcX	dsDNA	Nuc	Farr	CLIF
ACR criteria (ever), *N *= 207						
Renal	101	0.01	0.03	0.01	ns	ns
Neurologic	23	0.01	0.03	0.01	0.04	ns
Hematological	134	ns	ns	ns	ns	0.04
mSLEDAI 2000 (current), *N *= 165						
Casts	3	0.03	0.04	0.04	0.02	0.04
Hematuria	13	<0.001	0.001	0.003	0.002	ns
Proteinuria	16	0.04	ns	ns	ns	ns
Leukocyturia	3	0.04	0.02	ns	ns	ns
Pleuritis	5	ns	ns	ns	0.01	ns
Pericarditis	10	ns	0.02	ns	ns	ns
Complement	84	0.003	0.005	0.004	0.01	0.03
Fever	8	0.02	0.04	ns	ns	ns
Thrombocytes	5	ns	0.04	ns	ns	0.03
Laboratory (current), *N *= 165						
Decreased lymphocytes	91	0.02	0.02	ns	0.03	ns
Decreased monocytes	27	0.02	0.02	ns	ns	ns
Decreased C-reactive protein	62	0.02	0.04	0.04	ns	ns
Decreased C3	83	0.004	0.001	0.01	0.004	ns
Decreased C4	63	ns	0.04	ns	ns	ns
Proteinuria >150 mg/d	39	0.01	ns	0.04	ns	ns

^a^Comparison was performed using the Mann-Whitney *U *test. This table is reduced to statistically significant findings to increase readability. Significance with regard to any feature was always found for increased values of autoantibody assays. The number of patients with a positive finding (ACR and SLEDAI 2000) or an abnormal finding (local laboratory) of all patients is given. Current values refer to values on the date of blood withdrawal. NcX, anti-dsDNA-nucleosome-complexed enzyme-linked immunosorbent assay (ELISA); dsDNA, dsDNA, double-stranded DNA; Nuc, anti-dsDNA-nucleosome ELISA; Farr, radioimmunoassay; CLIF, *Crithidia luciliae *immunofluorescence assay; ACR, American College of Rheumatology; mSLEDAI 2000, modified Systemic Lupus Erythematosus Disease Activity Index 2000 [25]; C3, complement component C3; C4, complement component C4; ns, not significant.

Using the global mSLEDAI 2000 score (items used to score for anti-dsDNA and complements were excluded to avoid bias), only the Anti-dsDNA-NcX ELISA (*r*_*s *_= 0.145, *p *= 0.034) and anti-Nuc ELISA (*r*_*s *_= 0.143, *p *= 0.034) were significantly correlated on the basis of Spearman's rank order correlation Spearman's rank order correlation, but neither the anti-dsDNA ELISA (*r*_*s *_= 0.113, *p *= 0.074) nor the Farr assay (*r*_*s *_= 0.118, *p *= 0.065) showed a significant correlation.

All test systems were significantly associated with urinary casts and decreased complement component 3 (C3) at the time of blood sampling. Notably, some items were related to only one assay. So, the mSLEDAI 2000 items pericarditis and decreased complement component 4 (C4) were exclusively associated with elevated titers in the anti-dsDNA ELISA. The Farr assay was exclusively connected to the presence of pleuritis. The CLIF assay was associated with prior hematological manifestation according to an ACR criterion. It is noteworthy that the SLEDAI 2000 item proteinuria was solely associated with higher levels found by the Anti-dsDNA-NcX ELISA.

Of our 207 patients, 101 had histologically proven lupus nephritis. Of those 101 patients, 61 were not considered in further subgroup analysis because of a missing biopsy within 1 year before blood draw. Seven patients actually had lupus nephritis class II, 13 had class III, 12 had class IV and eight had class V according to the International Society of Nephrology Working Group on the Classification of Lupus Nephritis/Renal Pathology Society Working Group on the Classification of Lupus Nephritis guidelines [26]. Prior or present renal involvement (*n *= 101) was significantly associated with elevated test results in the anti-NcX ELISA (*P *= 0.002), anti-Nuc ELISA (*P *= 0.004), anti-dsDNA ELISA (*P *= 0.008) and the Farr assay (*P *= 0.042), but not in the CLIF assay (*P *= 0.812) (all *P *values based on the Mann-Whitney *U *test). Further analysis of defined classes revealed an exclusive correlation of lupus nephritis class IV class (*n *= 12) with elevated antibody levels on the basis of Anti-Nuc, Anti-dsDNA and Anti-dsDNA-NcX ELISA results using Spearman's rank order correlation (*r*_*s *_= 0.192, *r*_*s *_= 0.189, *r*_*s *_= 0.183, respectively; all *p *< 0.01).

### Association of assays with disease activity over time

**Figure 3:**
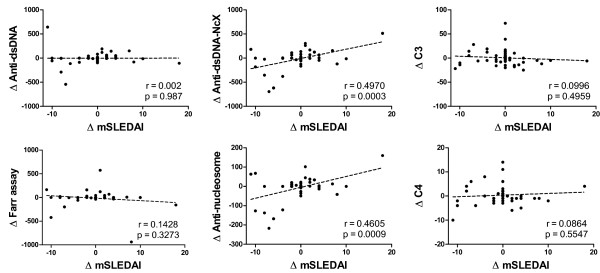
**Changes in disease activity versus changes of six defined laboratory parameters over time**. All results are based on a total of 69 patient visits of 20 different systemic lupus erythematosus patients. Delta values were calculated by subtracting values for a defined parameter from an actual visit from a defined parameter from the last visit (for example, ΔC3 = C3_(visit __*n *__+ 1) _- C3_(visit __*n*__)_. Only changes in Anti-dsDNA-NcX and antinucleosome ELISA results correlated significantly with changes in modified Systemic Lupus Erythematosus Disease Activity Index 2000 (mSLEDAI 2000 [25]) score over time. The mSLEDAI items for double-stranded DNA (dsDNA) and complement components C3 and C4 were excluded to avoid bias. Linear regression analysis was used to calculate significance.

Monitoring of disease activity is of prime importance in the clinical care of SLE patients. Regarding the correlation of anti-dsDNA antibodies with disease activity, contradicting reports have been published in the literature, ranging from a positive [8] over a missing [9] to a negative correlation [7]. In the longitudinal prospective approach over 10 months used in our present study, 20 SLE patients were monitored with an overall total of 69 patient visits resulting in 49 different data points. In all visits, no change in therapy within the past 4 weeks had occurred before medical records and blood were taken. To test whether changes in distinct laboratory parameters can reflect changes in disease activity, the anti-dsDNA ELISA, the Farr assay, the antinucleosome ELISA, the Anti-dsDNA-NcX ELISA, C3 and C4 were compared with the changes in mSLEDAI 2000 score over time (Figure 3). The mSLEDAI 2000 score was defined with an exclusion of items for dsDNA and complement components to avoid bias. In the follow-up study, none of the traditional biomarkers (anti-dsDNA ELISA, Farr assay or C3 or C4) were correlated with disease activity over time. However, Anti-dsDNA-NcX ELISA correlated best (*r *= 0.4970, *P *= 0.0003), followed by the antinucleosome ELISA (*r *= 0.4605, *P *= 0.0009) using linear regression. A subgroup analysis was not performed because of the limited number of patients.

## Discussion

In this study, the frequency of autoantibodies against dsDNA-complexed nucleosomes was investigated in different rheumatic diseases and healthy individuals and compared with levels of autoantibodies against dsDNA or nucleosomes alone. We have demonstrated that the use of dsDNA-complexed nucleosomes instead of dsDNA or nucleosomes individually as binding substrates is superior in ensuring the diagnosis of and assessing disease activity in SLE.

To comprehensively investigate the performance of the Anti-dsDNA-NcX ELISA, sera of 207 SLE patients, 357 disease controls and 400 healthy individuals were tested. A sensitivity of 60.4% and a specificity of 98.9% were calculated for the diagnosis of SLE at the manufacturer's threshold of 100 IU/ml. Only 8 (2.2%) of 357 disease controls with other rheumatic autoimmune diseases had elevated anti-dsDNA-NcX results. Whereas in six of these eight patients either the anti-dsDNA ELISA or the antinucleosome ELISA also revealed positive test results, none of these sera were positive in the Farr or CLIF assay. The Farr and CLIF assays also revealed false-positive test results, but not in those eight Anti-dsDNA-NcX ELISA-positive disease controls (Table 1).

In five anti-dsDNA-NcX false-positive disease controls, we found other circumstances potentially causing elevated autoantibody levels, such as minocycline, sulfasalazine, TNF inhibitors and an unknown infection with fever. Exclusion of these assumed five false-positive events resulted in an increased specificity of the Anti-dsDNA-NcX ELISA from 98.9% to 99.5%.

To further compare the performance of the Anti-dsDNA-NcX ELISA with other established test systems, a ROC curve analysis comprising 964 individuals was conducted. In that analysis, the Anti-dsDNA-NcX ELISA had the best performance among the investigated test systems. The superior performance criteria of the Anti-dsDNA-NcX ELISA, especially in direct comparison to the anti-dsDNA and antinucleosome ELISAs, resulted from the novel approach of utilizing dsDNA-loaded nucleosomes instead of dsDNA or nucleosomes alone. In addition, nearly 10% of all sera were positive in the Anti-dsDNA-NcX ELISA, but this was not the case in the anti-dsDNA ELISA or the antinucleosome ELISA, indicating that dsDNA-loaded nucleosomes are more consistent with the naturally appearing antigen.

Beyond these findings, comparative data from ROC curve analysis once more indicate that ANuA are also a highly frequent and very specific feature of SLE. Therefore, taking into account that ANuA arise earlier than anti-dsDNA antibodies [15], ANuA should be strongly considered as a criterion for the classification and diagnosis of SLE with the proviso that the determination be performed using a well-characterized test system with proven specificity.

By studying differences between assays, we also found that all investigated test systems were able to indicate a positive test result when another test reported a negative test result (Table 2). The Anti-dsDNA-NcX ELISA showed the potential to completely replace the anti-dsDNA ELISA and ANuA ELISA, but not the Farr or CLIF assay. In harmony with the superior performance criteria in ROC curve analysis, the Anti-dsDNA-NcX ELISA revealed the lowest percentages of sera positive in assays other than the Anti-dsDNA-NcX ELISA. Surprisingly, and in conflict with current clinical practice, there were some sera that were found to be positive in the CLIF assay but negative in another test system. Thus, to cancel the CLIF assay because another upstream test (for example, anti-dsDNA ELISA) delivered a negative test result might circumvent a correct diagnosis of SLE in some cases. To increase the likelihood of correct diagnosis of SLE in clinically suspected cases, it appears useful to order several assays and techniques in parallel, since there is still no test that detects all antibody specificities. The observed differences are most likely caused by the different techniques used in the investigated assays.

Analysis of clinical findings with test results of investigated assays in our SLE patient cohort revealed that increased titers were differentially associated with neurological, renal and hematological involvement according to ACR criteria. Moreover, high titers were preferentially associated with active lupus nephritis class IV, casts, hematuria, proteinuria, leukocyturia, leukocytopenia, monocytopenia and consumption of C3. The highest number of significant associations with clinical features (*n *= 14) was revealed by the anti-dsDNA ELISA, followed by the Anti-dsDNA-NcX ELISA (*n *= 13). Remarkable differences were found in this analysis between the anti-dsDNA ELISA, the Farr assay and the CLIF assay, all of which target anti-dsDNA. Underlying methodical differences (radioimmunoassay versus immunofluorescence assay versus ELISA) might contribute to that phenomenon.

All assays were significantly correlated to global disease activity (assessed by mSLEDAI 2000 score) in the cross-sectional survey. Using serial data of 20 patients (69 patient visits), we further assessed whether test systems are useful for monitoring disease activity over time. Surprisingly, changes in broadly accepted biomarkers were not significantly associated with changes in disease activity over time. These findings are in harmony with recent data derived from a much larger serial analysis of 1,116 patient visits [9]. Strikingly, both ELISAs containing nucleosomes as antigens were well correlated with disease activity over time. However, because of the limited number of patients and samples per patient, the results have to be confirmed in larger studies.

## Conclusions

The nonradioactive Anti-dsDNA-NcX ELISA, which is based on dsDNA-loaded nucleosomes as antigens, demonstrated excellent test characteristics for the assessment of the diagnosis and disease activity that can be optimized even if clinicians interpret delivered test results within a medical context and consider the presence of drugs and infections having the potential to induce autoantibodies against dsDNA and/or nucleosomes.

## Abbreviations

ACR: American College of Rheumatology; AUC: area under the curve; CLIF: *Crithidia luciliae *immunofluorescence; dsDNA: double-stranded DNA; ELISA: enzyme-linked immunosorbent assay; mSLEDAI 2000: modified Systemic Lupus Erythematosus Disease Activity Index; RA: rheumatoid arthritis; RIA: radioimmunoassay; ROC: receiver operating characteristic; SLE: systemic lupus erythematosus; SS: Sjögren's syndrome; SSc: systemic sclerosis.

## Competing interests

RB was employed from August 2006 until March 2009 by Charité University Hospital with third-party funds paid by EUROIMMUN Medizinische Labordiagnostika AG. CD and AR are employees of EUROIMMUN Medizinische Labordiagnostika AG, Lübeck, Germany. WSc and WSt are board members of EUROIMMUN AG. The other authors declare that they have no conflict of interest.

## Authors' contributions

KE, CD, GRB, FH and WSc designed the study. FB, RB, TR, MB, AB and AR acquired data.

RB and FH analyzed and interpreted the data. RB, GRB, WSc, WSc and FH prepared the manuscript. OJ, RB and AR performed statistical analysis. KE, CD, WSt, FH and WSc were responsible for overall project management. RB had full access to all of the data in the study and takes responsibility for the integrity of the data and the accuracy of the data analysis.

## Supplementary Material

Additional file 1 **Supplementary Table S1. Characterization of SLE patients**. SLEDAI, Systemic Lupus Erythematosus Disease Activity Index; ^1^Number of patients with fully accessible SLEDAI 2000 data [25]; ^2^SACQ, serologically active clinical quiescent. Click here for file
