# Supplementary material for: Anti-dsDNA-NcX ELISA: dsDNA-loaded nucleosomes improve diagnosis and monitoring of disease activity in systemic lupus erythematosus
Source: Arthritis Res Ther. 2011 Feb 10;13(1):R26. doi: 10.1186/ar3250 (PMC3241370; doi:10.1186/ar3250)
Supplement: Additional file 1 — Supplementary Table S1. Characterization of SLE patients. SLEDAI, Systemic Lupus Erythematosus Disease Activity Index; 1Number of patients with fully accessible SLEDAI 2000 data [25]; 2SACQ, serologically active clinical quiescent. [file ar3250-S1.DOC]

**Supplementary Table S1**

|  |  |  |
| --- | --- | --- |
|  |  |  |
| **Features** | | **SLE patients**  (n = 207) |
|  |  |  |
|  |  |  |
| Age, mean (range) years | | 40.0 (18-80) |
| Female, no. (%) | | 184 (88.9) |
| Ethnicity | |  |
|  | Caucasian, no. (%) | 200 (96.6) |
|  | Asian, no. (%) | 6 (2.9) |
|  | Afroamerican, no. (%) | 1 (0.5) |
| No. of ACR-criteria for SLE fulfilled mean (range) | | 5.4 (4-9) |
|  | Malar rash in % | 64.7 |
|  | Discoid rash in % | 07.2 |
|  | Photosensitivity in % | 39.2 |
|  | Oral ulcers in % | 15.0 |
|  | Arthritis in % | 73.2 |
|  | Serositis in % | 36.6 |
|  | Renal disorder in % | 52.3 |
|  | Neurologic disorder in % | 11.8 |
|  | Hematologic disorder in % | 43.1 |
|  | Immunologic disorder in % | 92.8 |
|  | Antinuclear antibody in % | 99.4 |
| SLEDAI 2k completed^1^, no. (%) | | 165 (79.7) |
|  | Mean (range) | 5.4 (0-26) |
|  | SACQ^2^, no. (%) | 41 (24.8) |
| Disease duration, mean (range) years | | 10.1 (0-40) |
|  |  |  |
|  |  |  |

Characterization of SLE-patients: SLEDAI = Systemic Lupus Erythematosus Disease Activity Index; ^1^ Number of patients with full-assessible SLEDAI 2000 [23]; ^2^ SACQ = Serologically Active Clinical Quiescent.
